# Prevalence and covariates of electrocardiographic left ventricular hypertrophy in diabetic patients in Tanzania

**Published:** 2008-02

**Authors:** JJK Lutale, H Thordarson, Z Gulam-Abbas, K Vetvik, E Gerdts

**Affiliations:** Institute of Medicine and Centre for International Health, University of Bergen, Bergen, Norway; Muhimbili University College of Health Sciences, Dar es Salaam, Tanzania; Department of Medicine, Haukeland University Hospital, Bergen, Norway; Muhimbili University College of Health Sciences and Abbas Medical Centre, Dar es Salaam, Tanzania; Institute of Medicine, University of Bergen, Norway; Institute of Medicine, University of Bergen, Norway

## Abstract

**Background:**

Left ventricular hypertrophy (LV H) has been demonstrated to be a powerful predictor of cardiovascular (CV) morbidity and mortality in diabetic as well as hypertensive patients. However, less is known about the prevalence of electrocardiographic LV H (ECG-LV H) and its relation to other CV risk factors in diabetic patients in sub-Saharan Africa. Therefore, the aim was to assess the prevalence of ECG-LV H in diabetic patients in Dar es Salaam, Tanzania, and its relation to other cardiovascular risk factors.

**Methods:**

Two hundred and thirty-seven consecutive patients attending the Muhimbili diabetic clinic were studied. ECG-LVH was diagnosed by Sokolow-Lyon voltage and Cornell voltage-duration product criteria. Q waves, ST-segment deviation, T-wave abnormalities and intraventricular conduction defects were classified by the Minnesota codes. Blood pressure (BP), serum creatinine, cholesterol and triglyceride levels, and HbA_1c_ and urinary albumin and creatinine concentrations were determined.

**Results:**

The prevalence of LV H in patients was 16% by either ECG criteria; 12.2% by Sokolow-Lyon and 5.1% by Cornell product criteria. Patients with LV H had significantly higher systolic and mean BP and pulse pressure, and a higher prevalence of ST-segment abnormalities, T-wave inversion and albuminuria than those without LV H (all *p* < 0.05). In multivariate logistic regression analysis, systolic BP was the only independent predictor of ECG-LV H. The prevalence of ECG-LV H increased by 15% per 10 mmHg higher systolic BP [OR 1.151 (95% CI 1.00921.314), *p* < 0.05]. Clustering of cardiovascular risk factors differed significantly between type 1 and type 2 diabetes patients. On average, type 1 patients had 0.8 and type 2 had 2.2 additional CV risk factors.

**Conclusion:**

ECG-LV H was present in 16% of diabetic patients in Tanzania. Systolic BP was the most important predictor of ECG-LV H. Clustering of CV risks was significantly higher in type 2 than in type 1 diabetics, demonstrating the need for systematic multiple risk-factor assessment in these patients.

## Summary

Left ventricular hypertrophy (LVH), whether diagnosed by electrocardiography or echocardiography, is a manifestation of cardiac target-organ damage and has been demonstrated to be a powerful predictor of cardiovascular morbidity and mortality in diabetic^1^,^2^ as well as hypertensive patients.^3^,^4^ Physiologically, LVH is a structural and functional adaptation of the left ventricle chamber to increased afterload. In previous studies, main determinants of ECG-LVH, including advanced age,^5^ male gender,^6^,^7^ obesity,^8^,^9^ glucose intolerance, diabetes mellitus, lipid abnormalities, cigarette smoking and micro-albuminuria^10^ have been identified.

ECG-LVH has particularly been associated with hypertension in African patients.^11^-^13^ However, less is known about prevalence of electrocardiographic left ventricular hypertrophy (ECG-LVH) and its relation to other cardiovascular (CV) risk factors in diabetic patients in sub-Saharan Africa. Therefore, the aim of the present study was to assess the prevalence of ECG-LVH and its relation to other CV risk factors in diabetic patients attending the diabetes outpatient clinic at the Muhimbili National University Hospital in Dar es Salaam.

## Methods

All 290 patients attending the diabetes outpatient clinic at Muhimbili National Hospital between 1 August 2003 and 1 February 2004 were invited to participate in the study. All 271 patients without cardiac or renal failure, cerebral vascular disease or advanced liver disease were invited; 263 accepted participation in the present study. Muhimbili Hospital is the national referral and a university teaching hospital. All patients gave written informed consent before enrolment in the study.

The study was approved by the Scientific and Publication Committee of Muhimbili University College of Health Sciences and the Regional Ethical Committee III in Norway. The study was conducted in accordance with the Helsinki declaration.

Patients were classified as type 1 or type 2 diabetics according to the 1997 World Health Organisation (WHO)^14^ clinical criteria, based on age at diagnosis, mode of onset (acute versus insidious presentation), duration of disease, current treatment, body mass index (BMI), waist-to-hip ratio, blood pressure, random or fasting glucose, HbA_1c_ and urine ketone levels. Patients aged 30 years or younger at onset of diabetes, with acute presentation of classical symptoms, who required insulin therapy to control hyperglycaemia were classified as type 1. Patients older than 30 years who needed insulin treatment, even if they had had diabetes for a short duration, or were metabolically uncontrolled and/or underweight, were also classified as type 1.

Patients over 30 years at diagnosis and not needing insulin for metabolic control were classified as type 2 diabetics. Patients younger than 30 years were also classified as type 2 if they were obese and/or had diabetes duration of more than 10 years without the need of insulin treatment. Patients not fitting into the clinical features of either type were classified as undetermined and excluded from the present analysis.

All patients completed a questionnaire on demographics and CV history including smoking behaviour, presence of hypertension and use of antihypertensive drugs. Clinic blood pressure was measured in the supine position using a calibrated mercury sphygmomanometer after at least half an hour of rest in a quiet room. The systolic BP (SBP) was recorded at Korotkoff phase one and diastolic BP (DBP) at phase five. Blood pressure was measured twice within an interval of five to 10 minutes; the second measurement was taken as the clinic BP.

Body weight was measured to the nearest 0.5 kg and height to the nearest 0.5 cm. Body mass index was calculated as weight (kg)/height (m^2^) and categorised according to the WHO physical status interpretation.^15^ Patients with a BMI of ≤ 18.5 kg/m^2^ were regarded as underweight, those with a BMI of 18.5–24.9 kg/m^2^ as normal, 25.0–29.9 kg/m^2^ as overweight and those ≥ 30 kg/m^2^ as obese. Waist and hip circumferences were measured in centimetres and the waist-to-hip ratio (WHR) was calculated. Waist circumference (WC) was measured to the nearest 0.1 cm, at the midway point between the lowest rib and the iliac crest, at minimal respiration. Men with WC above 102 cm (40 inches) and women with WC above 88 cm (35 inches) were considered to have abdominal obesity.^16^

## Electrocardiogram

A resting, standard 12-lead ECG was obtained in all patients in a supine position in a quite room. Recording was done at a speed of 25 mm/sec and calibration was standardised at 10 mm/mV on a Schiller electrocardiograph Cardiovit AT-1 (Schiller AG, model No: SHL41, Altgasse 68, CH-6341 Baar, Switzerland).

The rhythm was read from the rhythm strip obtained from lead V1. The rhythm was classified as sinus rhythm, atrial fibrillation or other. Left ventricular hypertrophy was diagnosed by Sokolow-Lyon voltage criterion (the sum of the amplitude of the S wave in lead V1 or V2 and R wave in lead V5 or V6, in both genders) and Cornell voltage-duration product criterion (the sum of the amplitudes of the R wave in lead aVL and S wave in lead V3, adding 6 mm in women, and multiplied by the QRS duration). LV hypertrophy was considered present if the Sokolow-Lyon voltage criterion was above 35 mm,^17^ or if the Cornell voltage-duration product criterion was above 2 440 mm/ms. 18-21 Q waves, (code 1-1), ST-segment deviation (codes 4-1 to 4-4), T-wave abnormalities (codes 5-1 to 5-4) and intraventricular conduction defects (codes 7-1 to 7-8) were classified using the Minnesota codes.^22^

## Blood and urine analysis

Blood samples were drawn from the antecubital vein, centrifuged and stored at −80°C until shipped to the central clinical laboratory at the Haukeland University Hospital in Bergen, Norway. Samples were analysed on a Modular Analytics SWA auto analyser (Roche Diagnostics GmbH, 68298 Mannheim Germany) using kits produced by Boehringer Mannheim (Mannheim, Germany). Serum and urine creatinine were analysed using a modified kinetic method of the Jaffé reaction, serum cholesterol was assessed on cholesterol oxidase p-aminophenazone (CHOD-PAP) levels, and serum triglycerides by the glycerol phosphate oxidase p-aminophenazone (GPOPAP) method.

Capillary blood glucose (fasting or random), haemoglobin (Hg) and HbA_1c_ levels were measured in Dar es Salaam. Capillary blood glucose was measured on a HemoCue AB glucose analyser (Angelholm, Sweden), haemoglobin on a HemoCue analyser and HbA_1c_ was assessed by DCA 2000®+ (Bayer Corporation). The DCA 2000®+ was standardised against the DCCT method and verified in 1996.^23^ Quality control was maintained using standardised solutions.

Urine analysis was done on two overnight specimens collected on two separate days. Samples were screened for features of urinary tract infection and excluded if present. Urine albumin concentrations were determined using an automated immunoturbidity assay with a sensitivity of 2.3 mg/l and inter- and intra-assay coefficients of variation of 4.4 and 4.3%, respectively. A urinary albumin excretion rate (AER) of ≤ 20 μg/min was categorised as normoalbuminuria, 20.1−200 μg/min as microalbuminuria and AER levels of > 200 μg/min as macroalbuminuria.

## Cardiovascular risk factors

The CV risk factors in this study were assessed and defined as described by the 1999 WHO/ISH guidelines.^24^ These included waist circumference, hypertension, ECG-LVH, albuminuria, advanced age, smoking, dyslipidaemia and albuminuria. Waist circumference was chosen as the indicator variable for abdominal obesity as it has been established to be a better predictor of cardiovascular health risks than BMI.^25^ Subjects were considered to be hypertensive when the clinic blood pressure was ≥ 140 mmHg systolic and/or ≥ 90 mmHg diastolic, or when using antihypertensive treatment.^26^ Advanced age was defined as age over 55 years for men and over 65 years in women. Dyslipidaemia was defined as total cholesterol levels of above 6.5 mmol/l and/or LDL cholesterol above 4.0 mmol/l, and/or HDL cholesterol below 1.0 mmol/l for men and below 1.2 mmol/l for women. Albuminuria was considered present if AER ≥ 20 μg/min. Diagnosis of ECG-LVH has been described in detail above.

## Statistical analysis

This was performed using the statistical package for social sciences (SPSS) software, version 13 for windows (SPSS, Inc, Chicago, Illinois). Continuous data are reported as mean ± two standard deviations and categorical data as proportions. For variables with skewed distribution, which were age, diabetes duration, systolic and diastolic BP, AER and creatinine clearance, the median ± range was used. Patients were grouped according to type of diabetes.

Groups were compared using the unpaired *t*-test, Fisher exact test and Mann-Whitney test where appropriate. A logistic regression analysis test was used to quantify the association between covariates and the presence of ECG-LVH. In regression analysis, results are given per 10 mmHg higher systolic BP, per 5 mmHg higher diastolic BP and per 40 msec longer QRS duration for meaningful clinical interpretation. Results of regression analyses are given as odds ratio (OR) with 95% confidence interval (CI). A two-tailed p-value less than 0.05 was considered statistically significant in univariate and multivariate analyses.

## Results

Two hundred and seventy-one patients were enrolled. Of these, 54.3% were women; six patients did not complete the study and were excluded, with 263 patients remaining. In 14 patients, the type of diabetes could not be clearly determined and they were excluded from the present study population, as were 12 patients (six type 1 and six type 2) who did not have an ECG taken. In the final study population consisting of 148 type 2 and 89 type 1 diabetics, type 2 patients were significantly older (*p* < 0.001), had longer duration of diabetes (*p* < 0.05), higher body mass index (*p* < 0.001), waist circumference and waist-hip ratio (*p* < 0.001), and included more patients with hypertension (Table 1).

**Table 1.** Demographic Characteristics And The ECG Findings In The Study Population Divided By Type Of Diabetes

*Variables*	*Type 1 diabetes*	*Type 2 diabetes*
Number (%)	89 (37.6)	148 (62.4)
Age at inclusion (years)	20.8 (4−45)	51.8 (23.5−85)***
Women, *n* (%)	44 (49.4)	83 (56.1)
Diabetes duration (years)	3.0(0−17)	4 (0−25)*
Smoking, *n* (%)	
Current smoker	2 (2.4)	4 (2.7)
Ex-smoker	1 (1.2)	25 (17)
Never smoked	82 (96.5)	118 (80.3)
BMI (kg/m^2^)	19.4 (3.9)	27.8 (4.7)***
Waist circumference (cm)	70.3 (13.2)	94.2 (14.5)***
Waist−hip ratio (cm)	0.86 (0.73)	0.94 (0.11)***
Proportion with hypertension, *n* (%)	9 (11.7)	78 (54.2)***
ECG findings		
Any LVH criteria	14 (15.7)	23 (15.5)
Sokolow-Lyon voltage LVH	14 (15.7)	15 (10.1)
Cornell voltage-duration product LVH	0	12 (8.1)**
T-wave inversion	30 (33.7)	38 (25.7)
ST abnormality	13 (14.6)	18 (12.2)
Intraventricular conduction defects	8 (9.0)	38 (26)***

Data expressed as median (min−max) and mean (SD) as appropriate. *p < 0.05, **p < 0.01, ***p < 0.001.

## Prevalence of ECG-LV H

In the total study population, 37 (15.6%) patients had LVH by either Sokolow-Lyon or Cornell product criteria. The prevalence of ECG-LVH by the Sokolow-Lyon criterion was 12.2% of patients in the total study population, and by the Cornell product criterion 5.1%. The prevalence of LVH by any criteria did not differ between type 1 and type 2 patients (15.7 vs 15.5%, ns) (Table 1).

All type 1 patients with LVH were identified by the Sokolow-Lyon voltage criterion. Among the 23 type 2 diabetic patients with ECG-LVH, 12 were identified by the Cornell voltage product criterion and 15 by the Sokolow-Lyon voltage criterion. Four patients were identified by both criteria. Sixteen (44.4%) hypertensive patients and 11 (29.7%) patients with albuminuria had ECG-LVH identified by either the Cornell voltage product criterion or the Sokolow-Lyon voltage criterion.

## Covariates of ECG-LV H

Type 2 patients with ECG-LVH by either criterion had significantly higher systolic and mean BP than patients without ECGLVH (Table 2). There were, however, no differences between all the covariates in type 1 patients with or without ECG-LVH. In logistic regression analysis in type 2 patients of both genders, with diabetes type, age, diabetes duration, waist circumference, systolic and diastolic BP and albuminuria as the independent variables, ECG-LVH was associated with albuminuria [OR 4.046 (95% CI, 1.517−10.796), *p* < 0.01], and higher systolic BP [OR 1.534 per 10 mmHg higher SBP (95% CI, 1.081−2.176), *p* < 0.01] in univariate analysis.

**Table 2.** Cardiovascular Risk Factors And Biochemical Characteristics In Study Patients With And Without ECG-LVH

*Variables*	*Type 1 diabetes*		*Type 2 diabetes*	
	*No LVH*	*LVH by any criteria*	*No LVH*	*LVH by any criteria*
Basic characteristics *n* (%)	75 (84.3)	14 (15.7)	125 (84.5)	23 (15.5)
Women, *n* (%)	38 (86.4)	6 (13.6)	70 (84.3)	13 (15.7)
Body mass index (kg/m^2^)	19.5 (4.1)	19.4 (2.5)	27.9 (4.6)	27.7 (5.4)
Waist circumference (cm)	69.7 (14)	74 (6.4)	95.1 (12.3)	94 (12.4)
Proportion with abdominal obesity *n* (%)	38 (88.4)	5 (11.6)	31 (93.9)	2 (6.1)
Blood pressure				
Systolic BP (mmHg)	103 (72−140)	110 (100−140)	134 (98−210)	150 (90−200)*
Diastolic BP (mmHg)	73 (50−110)	78 (50−83)	84 (60−120)	86 (68−136)
Mean BP (mmHg)	84 (12)	88 (8)	102 (15)	110 (20)*
Pulse pressure (mmHg)	35 (9)	41 (17)	53 (16)	61 (22)
Proportion with hypertension, *n* (%)	7 (77.8.)	2 (22.2)	59 (81.9)	13 (18.1)
Heart rate (bpm)	83 (17)	82 (15)	76 (15)	78 (10)
Biochemistry				
Total cholesterol (mmol/l)	4.4 (1.3)	4.6 (0.7)	5.15 (1.3)	5.4 (1.2)
HDL cholesterol (mmol/l)	1.2 (0.4)	1.2 (0.3)	1.2 (0.44)	1.24 (0.42)
LDL cholesterol (mmol/l)	2.5 (1.03)	2.7 (0.8)	3.1 (1.1)	3.3 (1.0)
Serum triglycerides (mmol/l)	1.5 (1.3)	1.6 (1.7)	1.5 (0.9)	1.4 (1.3)
Serum creatinine (mmol/l)	48.3 (14)	51 (14)	74 (25)	79.4 (30)
Creatinine clearance (ml/min)	129 (46.4−482)	138 (74.7−206.9)	106 (39.6−273.4)	96.5 (49.6−178.5)
Albumin excretion rate (μg/min)	5.8 (0.7−290)	4.98 (1.44−91)	4.96 (1.3−2000)	4.85 (2.3−2000)
Proportion with albuminuria, *n* (%)	9 (82)	2 (18)	17 (65.4)	9 (34.6)
HbA_1c_ (%)	14 (6.3− > 14)	13.8 (8.6− > 14)	9.9 (4.4− > 14)	8.0 (5.4− > 14)
Other ECG findings				
IVC defects, *n* (%)	7 (87.5)	1 (12.5)	31 (81.6)	7 (18.4)
ST abnormalities, *n* (%)	8 (61.5)	5 (38.5)	13 (72.2)	5 (27.8)
T-wave inversion, *n* (%)	24 (80)	6 (20)	19 (76.3)	9 (23.7)

Data expressed as median (min−max) and mean (SD) as appropriate. *p < 0.05, **p < 0.01, ***p < 0.001.

Including the significant variables in a multivariate model, systolic BP was the only independent risk factor for ECG-LVH. The risk of ECG-LVH increased by 15% per 10 mmHg higher systolic BP [OR 1.541 (95% CI, 1.089−2.185), *p* < 0.01] (Table 3). The prevalence of ECG-LVH was also higher in hypertensive patients with albuminuria compared to hypertensive patients without albuminuria.

**Table 3.** COV Ariates Of LVH By Either Criteria In Type 2 Diabetic Patients Identified By Logistic Regression Analysis

*Covariates*	*LVH by any criteria*		
	*Number with LVH (%)*	*Unadjusted (simple) OR (95% CI)*	*Adjusted (multivariate) OR (95% CI)*
Gender: male	10 (15.4)	1.0	
female	13 (15.7)	1.021 (0.417−2.504)	
Age at inclusion (years)	23 (15.6)	1.016 (0.978−1.056)	
Diabetes duration (years)	23 (15.5)	0.968 (0.884−1.060)	
Waist circumference (cm)	22 (15.4)	0.990 (0.955−1.026)	
Serum cholesterol (mmol/l)	23 (15.6)	1.131 (0.808−1.584)	
Serum creatinine (mmol/l)	23 (15.6)	1.007 (0.992−1.023)	
Systolic BP/10 mmHg	22 (15.3)	1.534 (1.081−2.176)**	1.541 (1.089−2.185)**
Diastolic BP/5 mmHg	22 (15.3)	1.131 (0.932−1.373)	
Hypertension: no	9 (12.5)	1.0	
Hypertension: yes	13 (18.1)	1.542 (0.614−3.874)	
Normal AER	14 (11.6)	1.0	
Abnormal AER	9 (34.6)	4.046 (1.517−10.796)**	

Independent covariates involved in the logistic multivariate regression analysis are SBP/10 mmHg and AER. *p < 0.05, **p < 0.01, ***p < 0.001

The same model was used separately in type 1 diabetic patients. As described earlier, all type 1 patients with ECG-LVH were diagnosed by the Sokolow-Lyon criterion. Using the same model in these patients did not identify any significant covariate of ECG-LVH.

In type 2 patients, 12 had ECG-LVH diagnosed by the Cornell voltage-duration and 15 by the Sokolow-Lyon criteria. Applying the same univariate logistic regression model on these two groups of patients separately, it identified female gender, higher age, increasing waist circumference, higher systolic and diastolic BP and presence of hypertension or albuminuria as covariates of ECG-LVH by the Cornell voltage-duration criterion (all *p* < 0.05). In a multivariate analysis, systolic BP/10 mmHg was identified as the strongest independent covariate, [OR 2.210 (95% CI, 1.395−3.504), *p* = 0.001], followed by female gender [OR 10.475 (95% CI, 1.272−86.274), *p* = 0.029]. In a similar model with ECG-LVH by the Sokolow-Lyon criterion as the dependent variable, albuminuria was the only significant covariate of ECG-LVH in both univariate and multivariate logistic regression analyses [OR 1.001 (95% CI, 1.000−1.002), *p* < 0.05].

**Fig. 1.**
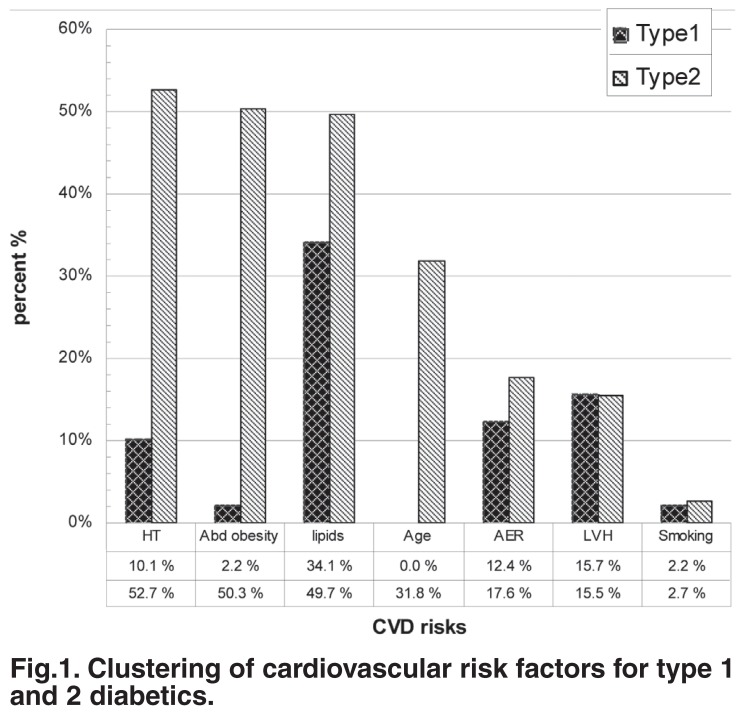
Clustering of cardiovascular risk factors for type 1 and 2 diabetics.

Clustering of the cardiovascular risk factors differed significantly between type 1 and type 2 diabetics. On average, type 1 patients had 0.8 (range 0−3) additional CV risk factor, while type 2 patients on average had 2.2 (range 0−6). In type 1 diabetics, dyslipidaemia in 30 (30.4%) and albuminuria in 11 (12.4%) patients were the most common additional CV risk factors. In type 2 patients, presence of hypertension was the most common additional CV risk factor present in 78 (54.2%) patients, followed by abdominal obesity, dyslipidaemia, albuminuria and advanced age (Fig. 1). In type 1 patients, 15% of these had one additional CV risk factor and 4.2% had two. Type 2 patients had up to six additional CV risk factors, 14.4% having one, 14% having two and 14.4% having three additional CV risk factors.

## Relation of other ECG findings with CV risk factors

In the overall study population, the prevalence of intraventricular conductance abnormalities was significantly higher in type 2 diabetics compared to type 1 patients (26 vs 9%, *p* < 0.001) (Table 1). The prevalence of T-wave inversion and ST-segment abnormality did not differ significantly between type 1 and type 2 patients (Table 1) and was significantly more common in patients with ECG-LVH by either criterion (Table 1).

The associations between prevalence of intraventricular conductance defects and other CV risk factors were assessed in a logistic regression model, including gender, diabetes type, age, diabetes duration, waist circumference, serum cholesterol, serum creatinine, systolic BP, diastolic BP, ECG-LVH, albuminuria and QRS duration among the covariates. In the univariate model, the presence of intraventricular conductance defects was associated with older age, longer duration of diabetes, higher systolic and diastolic BP and longer QRS duration (Table 4). In multivariate analysis, higher systolic BP was the only independent covariate of intraventricular conductance defects.

**Table 4.** COV Ariates Of Intraventricular Conduction Defects (IVC) In Diabetic Patients Identified By Logistic Regression Analysis In The Total Study Population

*Covariates*	*IVC yes n (%)*	*Unadjusted (univariate) OR (95% CI)*	*Adjusted (multivariate) OR (95% CI)*
Gender: male	23 (50)	1.0	
female	23 (50)	0.835 (0.438−1.591)	
Diabetes type 1	8 (9)	1.00	
Diabetes type 2	38 (25.7)	3.498 (1.549−7.899)**	
Age (years)	46 (19.6)	1.029 (1.011−1.049)**	
Diabetes duration (years)	46 (19.6)	1.070 (1.008−1.136)*	
BMI (kg/m^2^)	42 (18.6)	1.067 (1.009−1.129)*	
Serum cholesterol (mmol/l)	46 (20.1)	1.026 (0.869−1.216)	
Serum creatinine (mmol/l)	46 (20.1)	1.007 (0.995−1.018)	
SBP/10 mmHg	44 (20)	1.219 (1.077−1.378)**	1.221 (1.080−1.382)***
DBP/5 mmHg	44 (20)	1.180 (1.046−1.330)**	
LVH: no	38 (19)	1.0	
LVH: yes	8 (21.6)	1.176 (0.498−2.776)	
Normal AER	36 (78.3)	1.0	
High AER	10 (21.7)	1.636 (0.727−3.680)	
QRS duration/40 msec	46 (19.6)	3.526 (1.157−10.743)*	

Independent covariates involved in the multivariate analysis are diabetes type, diabetes duration, age, WC, SBP/10 mmHg and QRS/40 msec. *p < 0.05, **p < 0.01, ***p < 0.001.

In a similar model assessing covariates of ST-segment abnormality, female gender [OR 0.43 (95% CI, 0.2−0.9), *p* = 0.034], patients with ECG-LVH [OR 3.623 (95% CI, 1.456−9.015), *p* = 0.006] and diastolic BP/5 mmHg [OR 1.189 (95% CI, 1.012−1.397), *p* = 0.035] were identified as independent covariates of having ST-segment abnormality in a multivariate analysis. When T-wave inversion was the dependent variable in a similar model, the presence of ECG-LVH [OR 1.89 (95% CI, 0.9−3.9), *p* < 0.05) and age [OR 0.98 (95% CI, 0.969−1.000), *p* < 0.05) were associated with the presence of T-wave inversion in a univariate regression analysis.

## Discussion

This cross-sectional study was the first to investigate the prevalence and covariates of ECG-LVH in a group of diabetic patients in Dar es Salaam, Tanzania. The main findings from this study were a prevalence of 15.6% of subjects having ECG-LVH among the African diabetic patients, with median diabetes duration of three years; and secondly, identifying systolic BP and albuminuria as the main covariates associated with the presence of ECG-LVH in this study.

The prevalence of ECG-LVH among diabetic patients in sub-Saharan Africa is largely unknown; therefore the present study adds to previous knowledge. Most previous reports on the prevalence of ECG-LVH in Africans come from West Africa, where prevalence ranged from 4.2% using the Cornell voltage criterion among civil workers in Benin,^11^ to 22 and 48% using Cornell voltage and Sokolow-Lyon voltage criteria, respectively among hypertensive patients in Nigeria.^12^ A study from Kenya in newly diagnosed mild to moderate hypertensive patients found the prevalence of ECG-LVH to be 31.7%.^27^ Studies among African-American patients also showed high rates of ECG-LVH in hypertensives, 36.2% by Sokolow-Lyon and 23.4% by Cornell product criteria.^28^ The previous finding that more hypertensive African patients with ECG-LVH were picked by Sokolow-Lyon voltage than by Cornell product criteria is in accordance with findings in the present study.

Compared to results from the Eurodiab IDDM Complications study, the prevalence of ECG-LVH in type 1 diabetic patients in the present study was unexpectedly high.^29^ It is well known that the Sokolow-Lyon voltage criterion may overestimate the diagnosis of LVH in young, tall or thin subjects as included among the type 1 diabetic patients in our study. However, epidemiological studies in general East-African populations in the Republic of Seychelles reported ECG-LVH by the Sokolow-Lyon voltage criterion in 9.3% of patients.^30^ This study also found that the Sokolow-Lyon voltage criterion had low specificity for anatomical LVH in East African populations, suggesting that some race-specific ECG features may interfere with components of ECG-LVH diagnoses by the Sokolow-Lyon voltage criterion.

It is well known that CV risk-factor clustering in diabetic patients is associated with an increased risk for developing renal impairment and coronary vascular complications.^31^ In particular, patients with three or more risk factors are more likely to develop CV complications, such as coronary heart disease and stroke.^31^ Our finding that the prevalence of two or more cardiovascular risk factors was higher in type 2 than in type 1 diabetic patients (68.3 vs 15.7%, *p* < 0.001) is in accordance with previous findings, and underscores the necessity of broad screening for CV risk factors in type 2 diabetic patients at the time of diagnosis.^32^ In multivariate logistic regression, systolic BP and albuminuria were identified as the most important covariates of ECG-LVH.

In the current study, 16 (44.4%) of the patients with ECGLVH were also hypertensive. ECG-LVH among type 2 diabetics was associated with higher systolic and mean BP as well as the presence of hypertension, and all were significant covariates of the presence of LVH by the Cornell product criterion. However, with multivariate analysis, systolic BP [OR 1.015 (95% CI, 1.001−1.028), *p* < 0.05)] was the only independent covariate of ECG-LVH, while no independent association was found between diastolic BP and ECG-LVH. Similar findings have previously been reported from the LIFE study that included hypertensive patients with ECG-LVH by Cornell product or Sokolow-Lyon criteria.^33^ Likewise, in the Framingham study, patients with systolic BP > 180 mmHg had a 50% chance of developing ECG-LVH over 12 years, while no risk association was found with diastolic BP.^7^

Our finding, that hypertension and albuminuria were the main covariates of ECG-LVH is in accordance with previous reports in hypertensive patients and type 2 diabetics.^34^,^35^ Furthermore, Mbanya *et al.* demonstrated a significant correlation between left ventricular hypertrophy by echocardiogram and urinary albumin excretion rate among diabetic patients in Cameroun.^36^

In the current study, the prevalence of ECG-LVH was higher in type 2 diabetic patients with albuminuria than in normal-buminuric patients. Furthermore, the prevalence of ECG-LVH was also higher in hypertensive patients with albuminuria compared to hypertensive patients without albuminuria. The present study adds to previous knowledge by demonstrating a relationship between LVH, hypertension and albuminuria after a short duration of diabetes in Tanzanian type 2 diabetes patients. In the LIFE study, ECG-LVH using Cornell product or by Sokolow-Lyon criteria was associated with a 1.6-fold increase in the prevalence of microalbuminuria and a 2.6-fold increase in the prevalence of macroalbuminuria compared to those without LVH.^10^

In our study, ST-segment depression and T-wave inversion on ECG were associated with having ECG-LVH. The finding is in accordance with previous reports in hypertensive patients, finding strain patterns in the absence of coronary disease to indicate the presence of anatomical LVH.^37^,^38^ But such ECG findings can also indicate myocardial injury.^7^,^37^ However, in the present study population, there were no patients reporting clinical symptoms suggestive of coronary heart disease.

The median HbA_1c_ levels were high in both types of diabetics, indicating poor metabolic control. However, there was no correlation between HbA_1c_ and LVH in this study. Use of the Cornell product and Sokolow Lyon voltage criteria to diagnose ECG-LVH has been validated in large studies. However, the accuracy of the criteria used for detecting LVH has been found to vary with body size and gender. For instance, obesity decreased the sensitivity of the Sokolow-Lyon voltage ECG criterion^38^ and it was more sensitive in men, while the Cornell product criterion had greater sensitivity with women.^33^

Therefore, in the current population, these ECG criteria only partially identified different diabetic patients. Our findings are in agreements with previous reports from the LIFE study33 in which the above ECG criteria set for LVH also identified different hypertensive patients with different baseline characteristics. This finding further emphasises the importance of using more than a single criterion to more correctly diagnose patients with ECG-LVH.

## Study limitation

The major limitation of this study was that echocardiography was not performed to confirm anatomical LVH in patients with ECG-LVH. We chose ECG for diagnosing LVH because it is a more widely available, cheaper and more easily performed technique, albeit less sensitive and specific than echocardiography, for detection of LVH, in particular within African populations and in young or thin type 1 diabetic patients. However, previous publications from the LIFE study have documented that the majority of patients with ECG-LVH with the criteria used in our study indeed also had LVH detected by echocardiography.^33^ As the present study did not include a non-diabetic comparative group, the impact of diabetes alone on prevalence of ECG-LVH in the Tanzanian population could not be determined.

## Conclusion

This study demonstrates that ECG-LVH was present in 15.7% of type 1 and 15.5% of type 2 diabetic Tanzanian patients. Systolic BP and albuminuria were identified as the main predictors of the presence of ECG-LVH. Our study also demonstrated that CV risk factors cluster in type 2 diabetics, underscoring the need for broad screening of CV risk factors in these patients to optimise prevention of CV risk complications.
